# Beyond Total Content: Assessing the Bioaccessibility of Metals in Tattoo Inks Using Validated Extraction Media

**DOI:** 10.1002/ansa.70103

**Published:** 2026-08-07

**Authors:** Maria Luisa Astolfi, Lorenzo Zara, Arianna Antonucci, Carmela Protano

**Affiliations:** ^1^ Department of Chemistry Sapienza University of Rome Rome Italy; ^2^ MSD Animal Health Latina Italy; ^3^ Department of Public Health and Infectious Diseases Sapienza University of Rome Rome Italy

**Keywords:** bioaccessible fraction, CV‐AFS, ICP‐MS, tattoo inks, trace elements

## Abstract

A validated analytical workflow is presented for determining total and bioaccessible concentrations of regulated elements in tattoo inks, addressing the lack of harmonized protocols for comparing extraction approaches in complex matrices. Although current legislation defines limits for soluble fractions of selected metals, it does not specify the extraction media to be used, despite the heterogeneous composition of tattoo inks potentially affecting metal mobility and release. Total elemental concentrations were determined after microwave‐assisted acid digestion, while bioaccessible fractions were assessed using three in vitro extraction media: deionized water, artificial sweat and physiological saline. The analytical performance and reliability of the extraction procedures were validated. Elemental determinations were performed by ICP‐MS and CV‐AFS. The workflow showed good precision, reproducibility and comparability among extraction media, enabling systematic evaluation of matrix‐dependent metal release. Commercial inks exhibited high variability in elemental composition depending on colour and formulation. Lead exceeded regulatory limits in several samples. Yellow inks were enriched in Ba, Cd and Zn, whereas blue inks showed higher concentrations of Cu, As, Pb and Sn. Bioaccessibility strongly depended on the extraction medium (*p* < 0.05). Nickel reached 63% solubility in saline and 59% in artificial sweat in black inks, while Hg reached up to 72% in white inks. This approach links total elemental content to exposure‐relevant bioaccessible fractions and supports more harmonized safety assessment and regulatory monitoring of tattoo inks.

## Graphical abstract

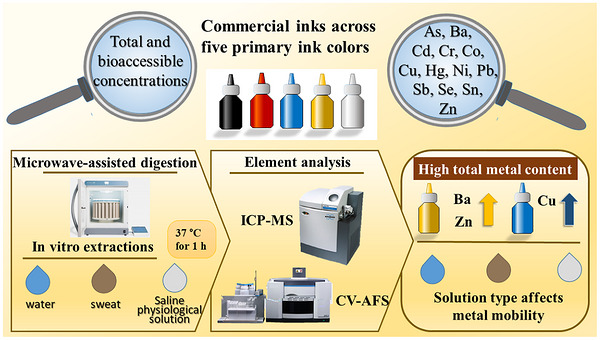

## 1 Introduction

Tattooing, once a cultural or ritual practice, has become a mainstream form of body art worldwide, with an estimated 10%–20% of the global population having at least one tattoo [1, 2, 3]. The growing popularity of tattoos has inevitably drawn scientific attention to the chemical safety of tattoo inks and permanent make‐up products and literature reported several health risks associated with tattooing and tattoo after‐care [4].

Tattoo inks are complex mixtures comprising organic and inorganic pigments, dispersants, solvents, binders, surfactants and various additives intended to stabilize the suspension or adjust viscosity [5]. However, alongside these functional ingredients, numerous studies have reported the presence of toxic metals and metal‐based nanoparticles—either as intentional pigment components, as by‐products of synthesis or as impurities introduced during manufacturing [6, 7, 8, 9]. Elements such as Cd, Co, Cr, Cu, Ni and Pb have been frequently detected in commercial inks [10]. The toxicological concern arises because the components of tattoo inks, once injected, are not inert. Metals and pigments can undergo partial dissolution, redox reactions and biotransformation within the dermal environment. Nanoparticles and soluble ions may be phagocytosed by macrophages, transported via the lymphatic system, and accumulate in lymph nodes or distant organs [11, 12]. Such processes underline the importance of assessing not only the total elemental content but also the bioaccessible fraction—the portion of an element that can potentially become available under physiological conditions [13, 14]. Bioaccessibility tests, which simulate dissolution in biological fluids such as artificial sweat, interstitial fluid or lysosomal extract, have thus become crucial tools for toxicological risk evaluation.

Concerns about the health impact of tattoo inks have prompted regulatory action, particularly in Europe. The most significant milestone is the Commission Regulation (EU) 2020/2081 [15], which amends Annex XVII of the REACH Regulation (EC) No 1907/2006 regarding substances in tattoo inks and permanent make‐up. This regulation introduces a new restriction (Entry 75) in REACH Annex XVII, establishing limits on the use of specific hazardous substances, including those classified as carcinogenic, mutagenic or toxic for reproduction (CMR Categories 1A and 1B), as well as skin sensitizers, irritants and substances corrosive to the eyes or skin [15]. The regulation sets maximum concentration limits for thousands of substances, including metals (e.g., Cr(VI), Ni, Cd, Pb, Hg, As, Sb), aromatic amines, and preservatives, and mandates the labelling of mixtures intended for tattooing with the statement ‘Mixture for use in tattoos or permanent make‐up’ along with the full ingredient list and batch identification. The general provisions became effective on 4 January 2022, with transitional allowances for pigments Blue 15:3 and Green 7 until January 2023. This regulatory step represents a major advance towards harmonized chemical safety standards for tattoo products in the European Union, filling a long‐standing gap previously covered only by national regulations or voluntary guidelines [2, 16]. Despite this progress, the implementation and enforcement of REACH‐based restrictions remain challenging. As highlighted by Bauer et al. [17] and Di Gaudio et al. [18], the analytical characterization of tattoo inks is hindered by their complex colloidal nature, strong pigment matrices and limited availability of validated analytical methods. Rubio et al. [5] further emphasized that, although analytical methodologies have improved in recent years, significant room for enhancing the analytical coverage of this field still remains.

The main challenge lies in developing a systematic analytical approach for the control of body‐decorating products, ideally supported by a clear and harmonized regulatory framework. Furthermore, commercial inks often contain pigment blends of variable composition, and even products marketed as ‘REACH‐compliant’ may still exceed concentration limits for certain metals or contain unapproved additives [6, 7]. According to current legislation [14, 15], only the soluble fraction of regulated elements such as Ba, Cu and Zn needs to be assessed; for Cu, ResAP(2008)1 [14] specifies that the soluble fraction should be determined after extraction into an aqueous solution at pH 5.5. However, no general standardized extraction medium or harmonized protocol is defined for the determination of soluble fractions across all regulated elements in tattoo inks.

The continuing detection of hazardous elements underlines the need for robust, standardized analytical workflows that can accurately quantify both total and bioaccessible metal fractions in these complex systems. Recent investigations have addressed this analytical challenge by developing high‐throughput and reliable workflows for multi‐elemental analysis of tattoo inks [18, 19].

The total elemental content is typically determined following microwave‐assisted acid digestion, which ensures complete decomposition of the organic matrix and dissolution of inorganic pigments. Common digestion media for total metal determination include concentrated nitric acid (HNO_3_), often combined with hydrogen peroxide (H_2_O_2_) or hydrochloric acid (HCl), under controlled pressure and temperature in closed‐vessel microwave systems. In cases where insoluble inorganic pigments such as TiO_2_, Cr_2_O_3_ or Fe_2_O_3_ are present, hydrofluoric acid (HF) is also added to achieve complete matrix decomposition and ensure quantitative metal recovery [6, 18, 19]. The resulting clear digests are then analysed using atomic spectrometric techniques, primarily inductively coupled plasma mass spectrometry (ICP‐MS) or inductively coupled plasma optical emission spectrometry (ICP OES), and less frequently by flame or graphite furnace atomic absorption spectrometry (FAAS or GFAAS) [6, 8, 19, 20]. In addition, Hg can be quantified by cold‐vapour atomic fluorescence spectrometry (CV‐AFS, also referred to as hydride generation AFS in cold‐vapour mode), which provides excellent detection limits for volatile mercury species and has recently been applied in tattoo and permanent make‐up ink analyses [7]. ICP‐MS is generally preferred for trace‐level determination of toxic elements such as As, Cd, Pb and Sb, whereas ICP OES offers robust quantification of major constituents (Cu, Cr, Fe, Mn and Zn). To assess bioaccessibility, inks are subjected to in vitro extraction tests simulating relevant biological environments [11, 13, 14, 21, 22, 23].

Typical extraction media include artificial sweat, artificial saliva and lysosomal fluid, prepared according to standardized compositions and maintained at physiological temperature (37°C) under continuous agitation [14]. After a defined contact time (usually 24–48 h), the suspensions are centrifuged or filtered (0.45 µm) to separate the soluble fraction, which is then analysed by ICP‐MS or ICP OES.

Comparison of the total and soluble concentrations provides the bioaccessibility percentage, offering a realistic measure of potential elemental release under physiological conditions. While total metal concentrations provide a general picture of ink composition, they often overestimate actual exposure risks. Metals tightly bound within insoluble pigment matrices may be poorly bioavailable and therefore less toxicologically relevant than those forming soluble or ionizable species [14]. Conversely, pigments containing transition metals can catalyse redox reactions and induce oxidative stress, potentially leading to inflammation and genotoxic responses [11, 13]. Integrating bioaccessibility assays with quantitative ICP analysis thus represents a more realistic and health‐relevant approach for risk assessment, aligning with emerging frameworks that encourage the use of in vitro and in silico new approach methodologies [24].

From a regulatory and analytical chemistry perspective, the combined determination of total and bioaccessible fractions provides data that are directly interpretable within the context of REACH restrictions [15]. Quantifying the portion of metals potentially available for systemic absorption allows authorities to better evaluate compliance and prioritize elements of concern. Moreover, recent studies have highlighted the need for continuous market surveillance and the development of standardized analytical protocols to ensure product safety [17, 19, 25]. A recently published study [23] investigated the dermally bioaccessible fraction of elements in tattoo inks using a sweat‐simulated extraction approach. While that work focused specifically on sweat extraction as a model of dermal exposure, the present study expands the analytical framework by comparing different validated extraction media, including deionized water, artificial sweat and physiological saline, in order to evaluate how extraction conditions influence metal solubility and bioaccessibility. In addition, the integration of total elemental analysis with comparative bioaccessibility assessment provides a broader evaluation of regulatory compliance and potential exposure relevance.

In light of these considerations, the present study aims to perform a comprehensive elemental analysis of tattoo inks by combining the determination of total and bioaccessible metal fractions using simulated biological fluids and spectrometric techniques. The proposed workflow integrates microwave‐assisted digestion for total elemental quantification with bioaccessibility testing, followed by ICP‐MS and CV‐AFS measurements. Particular attention was given to the soluble fractions of regulated elements such as Ba, Cu and Zn, whose assessment is required by legislation. However, no standardized extraction medium is currently prescribed for their determination, underscoring the importance of testing multiple aqueous solutions to evaluate possible differences in solubility. By comparing total and bioaccessible (or soluble) metal concentrations, this study aims to identify metals of regulatory and toxicological concern, assess compliance with the limits set out in REACH Annex XVII Entry 75 limits, and highlight the need for a harmonized analytical methodology for routine determination of the soluble fractions in tattoo inks.

## 2 Materials and Methods

### 2.1 Samples

A total of 110 tattoo inks from eight different brands and colour lines were purchased online. From this pool, a representative subset of 30 inks was selected, covering the five primary colours—black (6), white (6), red (6), yellow (6) and blue (6). These base colours were chosen because their pigments have distinct chemical compositions, and most other commercially available shades derive from their combinations. The selected inks were analysed to determine their elemental content following both microwave‐assisted acid digestion and extraction in different aqueous media. The products originated from China, the United States, and Austria, and included various types such as tattoo and microblading inks, permanent and semi‐permanent pigments, plant‐extract‐based and vegan formulations, and products labelled as non‐toxic or pure organic pigments. The inks, supplied in sealed containers with volumes ranging from 5 to 60 mL, were homogenized prior to analysis and stored in the dark at room temperature until sample preparation and digestion.

According to the product labels, the declared ingredients comprised mixtures of distilled or pure water, ethyl alcohol or ethanol, isopropanol or propylene glycol, glycerin or glycerol, witch hazel (*Hamamelis virginiana*) extract, and various pigments including C.I. 77266 (carbon black). Several inks also contained preservatives such as *Listerine* or unspecified stabilizers and claimed the use of natural or plant‐based colorants. These compositions are consistent with typical tattoo ink formulations, in which water, alcohols and polyols act as carriers facilitating pigment dispersion and skin penetration, while stabilizers, preservatives and pH regulators ensure product durability and microbiological safety [7].

### 2.2 Chemicals

All reagents were of high‐purity grade, suitable for trace elemental analysis. Concentrated HNO_3_ (suprapur, Carlo Erba Reagents, Milan, Italy), HCl (67%, super pure, Carlo Erba Reagents), HF (40% suprapure, Supelco, Merck KGaA, Darmstadt, Germany) and H_2_O_2_ (25%–35%, super pure for trace analysis, Sigma‐Aldrich, France) were used for sample digestion. Sodium borohydride powder (NaBH_4_, 98% purity; Merck KgaA) and NaOH anhydrous pellets (ACS‐ISO; Carlo Erba Reagents) were used to prepare the carrier and reducer solutions required for the CV‐AFS analysis. Deionized water was obtained by passing tap water through a Human Arioso Power I RO‐UP Scholar UV purification system (Human Bldg, Seoul, Korea). Artificial sweat (stock no. BZ496, purity 99.9%; pH ∼ 5.5; Biochemazone Inc., Canada) and physiological saline solution (0.9% NaCl; pH ∼ 5.5; Eurospital, Trieste, Italy) were used to simulate biological fluids for bioaccessibility assays. According to the manufacturer's specifications, the artificial sweat is a sterile ready‐to‐use solution formulated to mimic human sweat and contains electrolytes (Na^+^, K^+^, Ca^2+^, Mg^2+^, Cl^−^ and lactate), organic compounds (urea, lactic acid, ammonia and optional amino acids), an acetate‐ or citrate‐based buffering system and preservatives.

Multi‐element calibration standards (CPAChem, Bogomilovo, Bulgaria) containing 1 mg/L of As, Ba, Cd, Cr, Cu, Ni, Pb, Sb, Se and Sn in 5% HNO_3_, together with individual Zn (10 mg/L, 4% HNO_3_) and Co (1 mg/L, 4% HNO_3_) standards (CPAChem, Bogomilovo, Bulgaria), were used for external calibration. A Hg standard solution (1002 ± 7 mg/L; SCP Science, Baie D'Urfé, Canada) was used to prepare working standards for CV‐AFS analysis. Internal standards for ICP‐MS measurements were prepared at 0.005 mg/L by dilution of 1000 mg/L single stock solutions of Y (LGC Standards, VHG Labs, USA), In (Merck KGaA), Rh (Merck KGaA), Sc (Merck KGaA) and Th (Peak Performance CPI International, USA) in nitric acid (2%–7%). The isotopes Sc^45^, Y^89^, Rh^103^, In^115^ and Th^232^ were used as internal standards during all ICP‐MS measurements to minimize matrix‐induced variations, as they span a broad mass range and effectively compensate for fluctuations in sample introduction efficiency, plasma stability, matrix effects and instrumental drift [23, 26]. Certified reference materials (CRMs) used to verify the accuracy of the analytical procedures included NIST 1643f (Trace Elements in Water), NIST 1649a (Urban Dust) and SRM 2711a (Montana II Soil Moderately Elevated Trace Element Concentrations), all supplied by the National Institute of Standards and Technology (NIST, USA).

Graduated polypropylene vials (2.5–10 mL; Artiglass S.R.L., Due Carrare, Italy), syringes (Injekt, B. Braun Melsungen AG, Germany) and micropipettes (Gilson Inc., Middleton, USA) with disposable tips (EPT I.P.S. Standard Bulks, Hamburg, Germany) were used throughout the study. Syringe filters with membranes of hydrophilic polytetrafluoroethylene (PTFE) (0.45 µm; Merck KGaA), cellulose nitrate (0.45 µm; GVS Filter Technology Inc., IN, USA), cellulose acetate (0.20 µm; Lab Logistics Group, USA) and nylon (0.20 µm; Sartorius Stedim Biotech, Germany) were employed for sample filtration prior to instrumental analysis. All materials were pre‐cleaned with dilute nitric acid and rinsed with deionized water before use to minimize contamination.

### 2.3 Equipment

Sample weighing was performed using an analytical balance (Gibertini Europe 60, Italy). Controlled‐temperature treatments were carried out using a thermostatic water bath (WB 12; Argolab, Italy). Acid digestion of the tattoo inks was achieved using a microwave‐assisted digestion system (Ethos1 Touch Control; Milestone S.r.l., Sorisole, Bergamo, Italy) equipped with either PTFE or quartz digestion vessels, depending on the reagents used.

Multi‐elemental analysis was carried out by ICP‐MS (820‐MS; Bruker, Germany), equipped with a MicroMist glass nebulizer (0.4 mL/min; Analytik Jena AG, Germany) and a collision–reaction interface (CRI) cell used to reduce polyatomic interferences on isotopes such as ^52^Cr, ^5^ ^6^Fe, ^7^ ^5^As and ^7^ ^6^Se. For digests containing HF, a PFA‐Sapphire inert introduction kit (ESI Elemental Scientific Inc., Omaha, NE, USA) was employed to prevent corrosion and minimize memory effects. To verify optimal instrument performance, a standard solution containing 0.005 mg/L of Ba, Be, Ce, Co, In, Pb, Mg, Tl and Th was prepared daily in 1% HNO_3_ from a multi‐element stock solution (10.00 ± 0.05 mg/L; SpectroPure, Ricca Chemical Company, Arlington, TX, USA). The oxide content was monitored daily to ensure that the percentage of oxides (CeO^+^/Ce^+^) remained below 1%. For this purpose, the same standard solution was analysed each day, and instrumental parameters were optimized according to manufacturer specifications to achieve the best performance. The optimization criteria followed Bruker's recommendations, including oxide interference CeO^+^/Ce^+^ < 1%, doubly charged ion interference Ba^2+^/Ba^+^ < 3% and minimum sensitivity thresholds of ^2^ ^9^Be > 25,000 cps, ^115^In > 250,000 cps and ^232^Th > 100,000 cps. The following isotopes were monitored: As^75^, Ba^137^, Cd^112^, Co^59^, Cr^52^, Cu^65^, Ni^60^, Pb^208^, Sb^121^, Se^76^, Sn^118^ and Zn^66^. Detailed ICP‐MS operating conditions, including analyses performed with and without CRI for digests prepared with and without HF, are reported in Table S1.

Mercury was determined using a CV‐AFS instrument (AFS 8220 Titan; FullTech Instruments, Rome, Italy) operating in hydride‐generation mode with sodium borohydride as the reducing agent [27, 28]. The instrumental operating parameters for CV‐AFS are reported in Table S2. The NaBH_4_ solution (0.05% v/v, stabilized with 0.5% NaOH) was delivered at 3.6 mL min^−1^, whereas the HCl solution (5% v/v) was supplied at 2.5 mL min^−1^. The sample volume introduced into the system was 1.0 mL.

High‐purity Ar, H_2_ and He gases (purity > 99.999%; SOL Spa, Monza, Italy) were used as carrier and purge gases in both instruments.

### 2.4 Preparation of Tattoo Ink Samples

Ink samples were weighed using an analytical balance. Before sampling, each container was thoroughly homogenized by manual shaking, and the ink was withdrawn using a glass pipette equipped with a rubber bulb. The samples were subjected either to microwave‐assisted acid digestion for total elemental determination, using the microwave digestion program reported in Table S3, or to aqueous extraction procedures in simulated biological fluids (deionized water, physiological saline and artificial sweat) to assess the bioaccessible fraction of metals and metalloids.

Six method blanks were prepared for each analytical procedure using only the reagents, which underwent the same treatment steps as the real samples. Blank values were subtracted from all analytical results before data evaluation. The different sample pre‐treatments are described in detail in Sections 2.4.1 and 2.4.2.

#### 2.4.1 Microwave‐Assisted Digestion

Two microwave‐assisted digestion procedures were applied depending on the target analytes and the compatibility of the acid mixture with the analytical instrumentation. The multi‐element determination (all elements except Hg) was carried out using an HNO_3_/HF/H_2_O_2_ mixture, whereas Hg was quantified using an HF‐free procedure based solely on HNO_3_ and H_2_O_2_.

Method validation was performed for both HF‐based digestion and the HNO_3_‐based procedure used for Hg determination. Accuracy and precision were assessed using the CRM as well as fortified tattoo ink samples, with recovery tests conducted at 1/10 and 1/2 of the legal concentration limits (Table 1 and Table S4). Accurately weighed portions of approximately 100 mg of ink and 4 mg of certified material (NIST1649a) were transferred into PTFE vessels, and 3 mL HNO_3_, 1 mL HF and 1 mL H_2_O_2_ were added. Digestion was performed in a closed‐vessel microwave system according to the program reported in Table S3. Black and white inks, representing carbon black‐ and TiO_2_‐based formulations, were used for method optimization. After digestion, the solutions were diluted to 50 mL with deionized water and filtered through 0.45 µm PTFE syringe filters. Due to the presence of HF, these digests were analysed exclusively by ICP‐MS using an inert sample‐introduction system, after appropriate dilution (1:10, 1:100 or 1:1000). All samples were measured in triplicate.

**TABLE 1:** Certified values and analytical performance parameters for elements in NIST 1649a and SRM 2711a (for Hg) after total acid digestion.

Element	Certified value^a^	Experimental value^a^	Bias%^b^	RSD%^c^	LOD^d^	LOQ^e^
**As** ^f^	67 ± 2	54.9	−18	1.0	4	10
**Ba**	569 ± 21	530	−6.9	1.6	200	800
**Cd** ^g^	22	24.5	11	1.5	0.01	0.05
**Co**	16.4 ± 0.4	14.9	−9.0	6.4	0.2	0.6
**Cr** ^f^	211 ± 6	169	−20	0.3	20	60
**Cu**	223 ± 7	225	0.9	3.4	2	5
**Hg** ^h^	7.42 ± 0.18	6.77	−8.7	2.1	0.01	0.04
**Ni**	166 ± 7	161	−2.9	9.5	2	6
**Pb**	12,400 ± 400	13,000	4.6	3.8	2	7
**Sb**	29.9 ± 0.7	25.7	−14	4.9	0.1	0.3
**Se** ^f^	25.5 ± 0.7	25.0	−2.2	15	6	20
**Sn**	56 ± 13	54.8	−2.1	1.4	0.2	0.5
**Zn**	1680 ± 40	1670	−0.3	4.9	60	200

^a^ Concentrations are expressed in mg/kg.

^b^ Trueness bias percentage (*n* = 3).

^c^ Relative standard deviation (precision as repeatability, *n* = 3).

^d^ Limit of determination (mg/kg).

^e^ Limit of quantification (mg/kg).

^f^ The collision‐reaction interface (CRI) mode was applied for Cr, As and Se to minimize polyatomic interferences.

^g^ Cadmium value is provided as an information value only, as specified in the NIST 1649a certificate of analysis.

^h^ Element determined by cold vapour atomic fluorescence spectrometry (CV‐AFS).

To avoid the use of HF—an aggressive acid that can damage instrumentation and materials—an alternative digestion protocol based solely on HNO_3_ and H_2_O_2_ was evaluated for Hg determination by CV‐AFS [27, 28]. The procedure was validated using both fortified tattoo ink samples and the CRM SRM 2711a (Montana II Soil). Accurately weighed portions of sample were transferred into quartz digestion vessels: approximately 0.1 g of ink or 20 mg of SRM 2711a. Three independent replicates were prepared for each sample, starting from the weighing step, in order to account for the entire analytical procedure. Each sample was treated with 2 mL of HNO_3_ and 1 mL of H_2_O_2_, and digestion was performed in a microwave system equipped with a 20‐position rotor, following the temperature program reported in Table S3. After digestion was completed, vessels were allowed to cool for 20 min under a fume hood, and the solutions were quantitatively transferred into polypropylene tubes, diluted to 10 mL with deionized water and filtered through 0.45 µm cellulose nitrate syringe filters.

For CV‐AFS determination, all digests were adjusted to the required 3% HCl concentration. Digests of SRM 2711a were further diluted 1:20 to reach this concentration, whereas ink digests were acidified by adding 200 µL of HCl to 2 mL of digest.

#### 2.4.2 Bioaccessible Fraction (Simulated Fluid Extraction)

The bioaccessible elemental fraction (defined as the portion of an element likely to enter systemic circulation following dermal exposure) was determined using three aqueous extraction media: deionized water, physiological saline (0.9% NaCl) and artificial sweat. For each ink sample, accurately weighed aliquots (∼250 mg) were mixed with 5.0 mL of the respective extraction fluid and incubated in a thermostated water bath at 37°C for 1 h, following the procedure of Wang et al. [13] with a reduced extraction time to simulate initial metal release. While ResAP (2008)1 [14] recommends extraction of soluble Cu at pH 5.5, it does not specify an extraction duration; the selected incubation provides a reproducible estimate of the bioaccessible fraction within a physiologically relevant timeframe [13]. After incubation, samples were filtered sequentially through 0.45 and 0.20 µm syringe filters to remove suspended particles. Extracts obtained with physiological saline and artificial sweat were diluted 1:10 with 1% HNO_3_ prior to ICP‐MS analysis to prevent matrix interferences caused by high NaCl concentrations. High chloride content can lead to the formation of polyatomic species such as ^40^Ar^35^Cl^+^, which interferes with the determination of As (^7^ ^5^As^+^), and other argon‐ or sodium‐based ions (e.g., ^40^Ar^3^ ^7^Cl^+^, ^23^Na^52^Cr^+^) that may affect the accurate quantification of elements such as Se (^7^ ^8^Se^+^, ^8^ ^0^Se^+^), V (^51^V^+^) and Cr (^52^Cr^+^). These spectral overlaps can result in overestimated signals or increased background noise, compromising analytical accuracy. Dilution also reduces the overall total dissolved solids (TDSs) content, minimizing matrix‐induced signal suppression and preventing salt deposition in the sample introduction system, as recommended by the instrument manufacturer and established analytical guidelines [26, 29].

For CV‐AFS analysis of Hg, 2 mL aliquots of each extract were acidified with HCl to achieve a final concentration of 3% HCl.

To assess possible matrix interferences and verify analytical accuracy, the CRM NIST 1643f (trace elements in water) was diluted 1:10 in each of the three extraction media. For physiological saline and artificial sweat, the media had previously been diluted 1:10 with deionized water to match the treatment of the real ink extracts, while deionized water was used as is. The resulting solutions were analysed under the same conditions as the real samples. For each extraction fluid, six method blanks were prepared using only the reagents and subjected to the same treatment as the samples; the mean blank value was subsequently subtracted from all analytical results before data evaluation.

#### 2.4.3 Quality Control and Accuracy Assessment

The analytical performance of the methods was evaluated in terms of precision, accuracy, linearity, sensitivity, limit of detection (LOD), limit of quantification (LOQ) and instrumental drift.

Calibration curves for each element were prepared in the same matrix as the digested or extracted samples (deionized water, physiological saline 1:10 and artificial sweat 1:10) to ensure matrix‐matched quantification. Linearity was assessed over a concentration range encompassing the expected variation in the samples, with eight‐point calibration curves for ICP‐MS (0.1, 0.5, 1, 2, 5, 10, 20, 50 µg/L for most elements; Zn concentrations 10 times higher) and Hg (0.02, 0.04, 0.1, 0.2, 0.4, 0.8, 1.0, 1.5 µg/L). The linearity of each calibration was verified using the instrument software, and a minimum correlation coefficient (*R* ^2^) of 0.999 was considered acceptable. The LODs and LOQs were calculated as 3 and 10 times the standard deviation (SD) of replicate blank determinations, respectively. Accuracy and intra‐day precision were evaluated through the analysis of CRMs (NIST 1649a, SRM 2711a and NIST 1643f) and repeat measurements of both reference materials and fortified tattoo ink samples, respectively. Since NIST 1643f does not contain Hg and Sn, these two elements were added at intermediate concentrations relative to the calibration range (0.4 and 5 µg L^−1^, respectively) to allow recovery assessment.

Instrumental drift was monitored for both ICP‐MS and CV‐AFS by measuring a calibration standard after the initial calibration, every 20 samples, and again at the end of the analytical sequence. For ICP‐MS, the standard corresponded to the fifth calibration point, whereas for CV‐AFS the Hg standard at 0.4 µg/L was used. Drift (%) was calculated as [(*C_n_* − *C_i_*)/*C_i_*] × 100, where 𝐶_𝑖_ is the concentration immediately after calibration and 𝐶_𝑛_ is the measured concentration during the sequence. A maximum drift of ±10% was accepted; corrective actions, including instrument re‐optimization or recalibration, were applied when this threshold was exceeded. The last 20 samples preceding a drift excursion were re‐analysed. Furthermore, potential matrix effects and instrumental drift arising from temperature fluctuations, progressive cone obstruction or other instability sources during ICP‐MS analysis were corrected using a suite of internal standards (Sc, Y, Rh, In and Th), added to all standards, blanks and samples.

### 2.5 Data Elaboration

Statistical analyses were performed using Excel and SPSS Statistics 27 software (IBM Corp., Armonk, NY, USA). Non‐parametric tests (Kruskal–Wallis and pairwise post hoc tests) were applied to evaluate significant differences in the concentrations of elements extracted with the three different extracting solutions (deionized water, physiological saline and artificial sweat), as the data were not always normally distributed and the number of samples per group was unequal [30]. A *p*‐value lower than 0.05 was considered statistically significant.

For each element, the percentage of bioaccessible fraction (BF, %) in the aqueous extracts (deionized water, physiological saline and artificial sweat) was calculated relative to the total content determined after complete acid digestion. The BF% was computed using the following equation:

$$\text{BF}\%=C_{\text{extract}}/C_{\text{total}}\times 100$$

where *C*_extract_ is the concentration (mg/kg) of the element measured in the aqueous extract and *C*_total_ is the corresponding total concentration (mg/kg) obtained after microwave‐assisted acid digestion. The resulting percentages represent the portion of each element potentially available for solubilization under simulated physiological conditions, thus providing an estimate of their potential bioaccessibility and mobility once injected into the dermal matrix.

## 3 Results and Discussion

### 3.1 Method Performance

Due to the absence of a CRM specific for tattoo inks, method accuracy was assessed using CRMs of similar matrices (NIST 1649a, NIST 1643f and SRM 2711a for Hg in digests) and by analysing fortified ink samples. The validation parameters for total digestion and bioaccessibility analyses, summarized in Tables 1 and 2, Table S4, demonstrate that the methods achieved the required performance for multi‐element quantification in tattoo inks. Although a direct comparison with the literature is often difficult because analytical performance characteristics are not always comprehensively reported, and are not consistently available for all the elements investigated in the present study, the recoveries, precision, LODs and LOQs obtained here are generally comparable to those reported in recent investigations on tattoo inks and related matrices [7, 8, 18, 31, 32, 33, 34], confirming the reliability of the proposed methodology.

**TABLE 2:** Accuracy (expressed as bias% or recovery, R%) and intra‐day precision (RSD%) obtained for the elements measured in NIST 1643f (Trace Elements in Water) after 1:10 dilution in the three extraction matrices (deionized water, physiological saline and artificial sweat).

Elements	As	Ba	Cd	Co	Cr	Cu	Hg^a^	Ni	Pb	Sb	Se	Sn^a^	Zn
Deionized water													
**LOD** ^b^	0.01	0.03	0.00003	0.0002	0.03	0.006	0.00003	0.02	0.001	0.005	0.007	0.0002	0.2
**LOQ** ^b^	0.02	0.09	0.0001	0.0006	0.1	0.02	0.0001	0.07	0.005	0.016	0.02	0.0005	0.8
**bias or R%**	12	−1.0	5.9	6.4	13	13	90	16	7.0	5.9	5.0	90	20
**RSD%**	7.9	3.0	6.0	3.1	6.3	6.0	2.3	1.0	3.0	3.0	15	9.0	6.0
**Physiological saline solution**													
**LOD** ^b^	0.02	0.1	0.0004	0.0003	0.3	0.1	0.0004	0.03	0.003	0.0006	0.08	0.0002	0.3
**LOQ** ^b^	0.06	0.5	0.001	0.001	1	0.4	0.001	0.09	0.01	0.002	0.3	0.0006	0.8
**Bias or R%**	5.0	−2.0	11	−2.5	12	2.4	90	5.4	2.2	13	−18	100	9.0
**RSD%**	5.0	7.5	3.1	2.2	2.7	12	2.0	12	2.9	1.2	13	13	1.0
**Artificial sweat solution**													
**LOD** ^b^	0.02	0.03	0.002	0.002	0.2	0.07	0.001	0.04	0.01	0.001	0.04	0.0007	2
**LOQ** ^b^	0.06	0.1	0.005	0.005	0.6	0.2	0.004	0.1	0.02	0.004	0.1	0.002	7
**Bias or R%**	10	−13	18	−4.1	−10	12	94	−3.2	9.3	11	17	99	−3.6
**RSD%**	2.2	4.3	5.1	1.7	1.4	1.7	4.0	4.1	1.3	4.4	2.0	9.5	5.0

^a^ Hg and Sn are not present in NIST 1643f. For these elements, the reference material was fortified at intermediate concentrations relative to the calibration range (0.4, and 5 µg L^−1^, respectively), and recoveries (R%) were evaluated accordingly.

^b^ LOD and LOQ, limits of detection and quantification, respectively, are expressed as mg/kg.

#### 3.1.1 Total Digestion

The use of CRMs (NIST 1649a, and SRM 2711a for Hg in digests) confirmed the trueness of the digestion procedure, with most elements showing biases within ± 15% and precision consistently below 10% RSD (Table 1). For Se, a slightly higher RSD (∼15%) was observed, which is considered acceptable given its known analytical challenges in ICP‐MS and the complexity of the CRM matrix. Overall, the method meets commonly accepted performance criteria for ICP‐MS biomonitoring applications [35]. Larger negative deviations (> 15%) observed for some elements in the analysis of the CRM likely reflect the analytical challenges associated with complex heterogeneous solid matrices and matrix‐dependent elemental behaviour. Spike‐recovery tests performed on representative black and white inks further confirmed the method's accuracy, with most recoveries falling within the 85%–115% acceptance range. However, Zn, Sn and Pb showed recoveries slightly outside this interval. Precision was generally satisfactory (RSD% < 15%), although slightly higher values were observed for As, Sb and Se. Overall, the method can be considered robust, with acceptable performance within a ± 20% range for both recovery and precision. These results demonstrate that the method is suitable even for inks characterized by a complex pigment matrix (Table S4).

#### 3.1.2 Extraction of Bioaccessibility Elements

Accuracy in the extraction experiments was evaluated through the analysis of NIST 1643f diluted in each extraction medium, where recoveries generally fell within the expected ranges despite the diverse chemical properties of deionized water, physiological saline and artificial sweat (Table 2). As anticipated, LOD and LOQ values were lowest in deionized water and slightly higher in saline and sweat, reflecting their increased ionic strength and associated blank variability. Matrix‐dependent effects were most evident for elements prone to speciation changes, such as Se, although overall analytical performance remained robust. Precision remained within acceptable limits for all intra‐day assessments (RSD% < 15%, Table 2 and Table S4), confirming the good repeatability of both digestion and extraction workflows.

Taken together, the validation results demonstrate that the analytical workflow is suitable for the reliable determination of both total and bioaccessible metal fractions in tattoo inks, despite the complexity of the matrices and the variability among extraction media. This robustness supports its applicability for regulatory evaluation and contributes to the development of systematic analytical approaches for the control of body‐decorating products.

### 3.2 Total and Bioaccessible Elemental Concentrations

The concentrations of the total digested samples and their bioaccessible fractions in deionized water, physiological saline 1:10 and artificial sweat 1:10 are summarized in Tables S5–S9 and Table 3. It is important to note that several elements in Tables S5–S9 were only partially quantifiable across inks and extraction media. In particular, As, Cd, Co, Sb, Se and Sn frequently appeared below LOD or between LOD and LOQ in specific colours or extraction solutions, whereas Ba, Cu, Ni, Pb and Zn were consistently above LOQ in most matrices. The observed detection pattern—where several toxicologically relevant elements were quantifiable in total digests but frequently remained below LOD or LOQ in bioaccessible extracts—primarily reflects the intrinsically limited solubility of many pigment‐associated metal species under the tested aqueous and physiological conditions.

**FIGURE 1:**
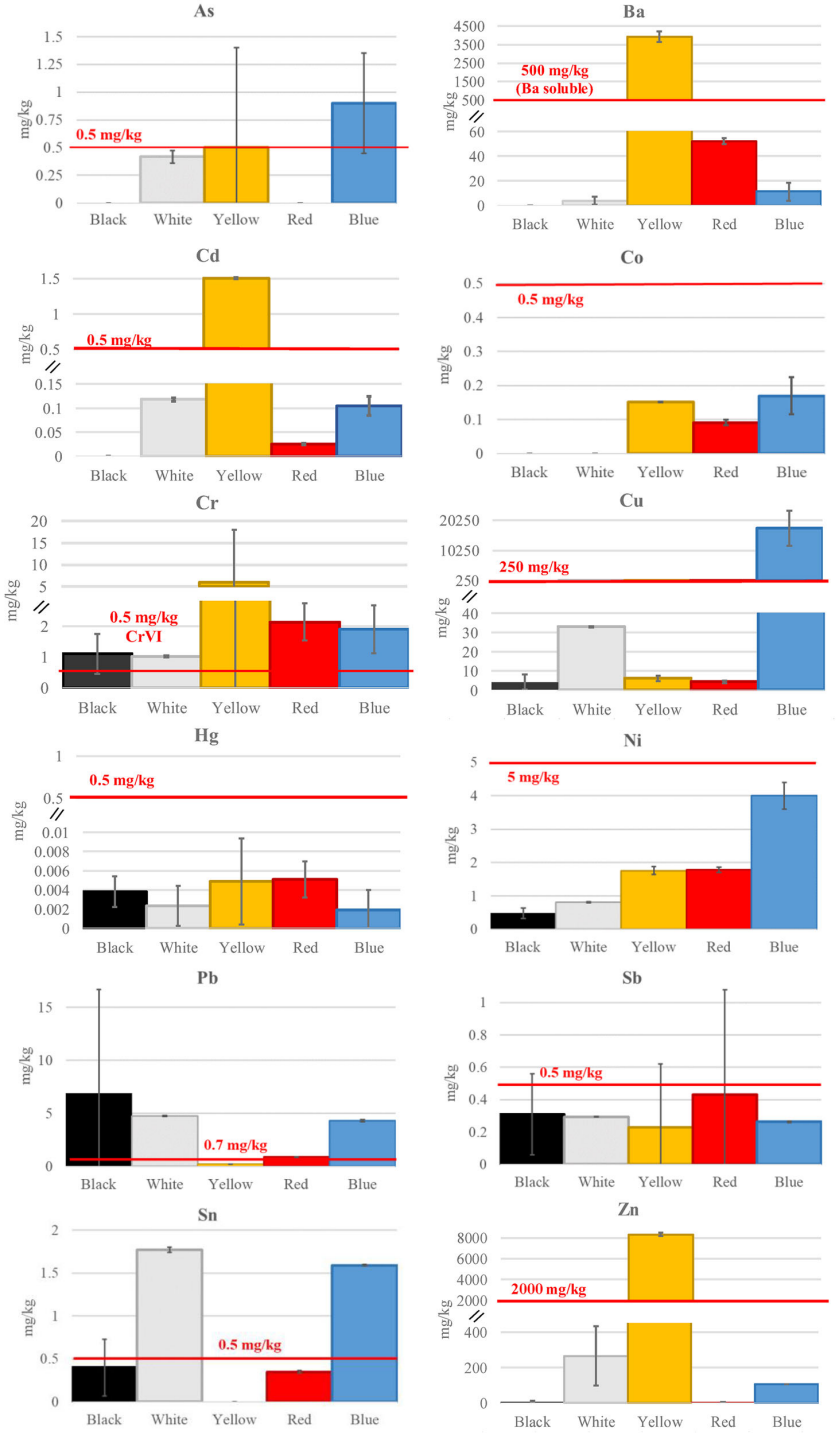
Element concentrations measured in ink samples (*n* = 6) after microwave‐assisted total acid digestion. Error bars represent the standard deviation (SD). Where applicable, the *y*‐axis includes a scale break (indicated by break marks) to facilitate the visualization of elements spanning a wide concentration range. The red line and red label indicate the regulatory limit set by Commission Regulation (EU) 2020/2081 (REACH).

As shown in Figure 1, the total acid digestion revealed a highly heterogeneous elemental composition among ink colours, confirming the intrinsic variability of pigment formulations reported in previous surveys of the European market [7]. As expected, Cu showed the largest variability, with concentrations up to the g kg^−1^ level in blue inks, consistent with the extensive use of Cu‐phthalocyanine pigments [18, 32]. Barium was systematically elevated in white, and yellow inks. It is extremely low solubility in all extraction media is consistent with the presence of sparingly soluble Ba salts, most plausibly BaSO_4_, which is widely used as a filler and whitening component in tattoo pigments. Cobalt fell below the LOD (< 0.004 mg/kg) in some samples. Similar occurrence patterns have been described in previous analytical surveys of tattoo inks [18, 32].

A comprehensive comparison with REACH limits [15] revealed that several inks exceeded regulatory thresholds for multiple elements. Total Cr concentrations were elevated across all colours; however, current legislation specifically applies to Cr(VI), and therefore compliance with the regulatory limit cannot be assessed using total Cr measurements alone. Nevertheless, literature data indicate that the proportion of Cr(VI) relative to total Cr can vary widely, ranging from 15% to 99% with a median of 45%, suggesting that the potential presence of Cr(VI) cannot be excluded [36]. Lead exceeded the regulatory limit in black, white, red and blue inks, despite its low bioaccessible fraction (≤ 2%). Yellow inks showed particularly elevated levels of Ba, Cd and Zn, whereas blue inks exhibited critical concentrations of As, Pb and Sn. Although measured Ni levels complied with REACH limit (5 mg/kg, Table S4), its frequent presence supports earlier observations of widespread but variable Ni occurrence below regulatory thresholds [7]. Total Pb concentrations exceeded the REACH limit of 0.7 mg kg^−1^ (Table S4) in black (6.9 ± 9.8 mg kg^−1^), white (4.75 ± 0.05 mg kg^−1^), blue (4.32 ± 0.08 mg kg^−1^) and red inks (0.887 ± 0.012 mg kg^−1^), while remaining below the regulatory threshold in yellow inks (0.216 ± 0.018 mg kg^−1^). Despite these elevated total contents, Pb exhibited very low bioaccessibility, with soluble fractions ≤ 2% across all extraction media. This limited solubility suggests that Pb is predominantly present in poorly soluble forms, likely associated with pigment matrices or inorganic additives, which restrict its immediate mobilization under simulated exposure conditions. Nevertheless, the detection of Pb at total concentrations exceeding regulatory limits remains a concern, as even small bioaccessible fractions may contribute to cumulative exposure over time. This aspect is particularly relevant given the well‐documented toxicity of Pb, which has been associated with severe long‐term health effects and poses a significant risk to foetuses, infants and children due to the vulnerability of the developing nervous system, as highlighted in previous studies [1, 32]. From a regulatory perspective, these findings emphasize the importance of considering both total and bioaccessible Pb fractions when assessing tattoo ink safety, as low solubility does not necessarily preclude potential health risks associated with chronic or repeated exposure.

**FIGURE 2:**
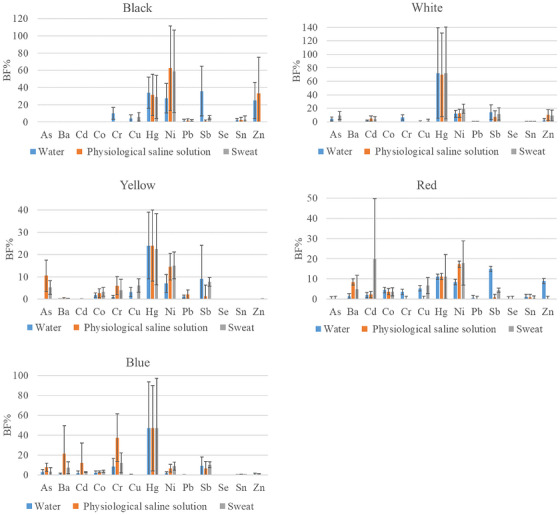
Percentage of bioaccessible fraction (BF, %) of the analysed elements in aqueous extracts obtained using deionized water, physiological saline solution and artificial sweat. Values represent the fraction of each element solubilized relative to its total concentration in the corresponding digested ink samples. Data are reported as mean ± standard deviation (SD).

The extraction experiments revealed marked differences among the three solutions—deionized water, physiological saline and artificial sweat—indicating that metal solubility is strongly modulated by the ionic strength and ligand composition of the medium (Tables S5–S9). Among the tested media, artificial sweat represents the most realistic scenario for dermal exposure, as it mimics the pH and ionic strength of human perspiration. However, it should be noted that artificial sweat lacks biological components such as proteins and lipids, which may affect metal binding and mobility in vivo. Deionized water and physiological saline, while useful for comparative purposes, represent simplified systems and may underestimate or overestimate metal release depending on the element and matrix. Overall, the choice of extraction medium should be guided by the intended exposure scenario, with artificial sweat being recommended for dermal risk assessment, while saline may better approximate interstitial fluid conditions. The bioaccessible fraction was low for most elements (typically < 1%–5%), although Cu, Zn and Ba displayed matrix‐dependent solubility increases in specific inks. Similar trends were previously described in artificial sweat dissolution studies, where complexation by lactic acid or chloride promoted partial release of Cu and Zn from pigmented matrices [32]. Barium generally exhibited low solubility across the extraction media, consistent with its occurrence in sparingly soluble forms such as BaSO_4_ or other Ba‐containing compounds; however, a higher bioaccessible fraction was observed in physiological saline for the blue ink (21%; Table S9), indicating that matrix composition and solution chemistry can significantly influence Ba mobilization. Conversely, appreciable Cu dissolution in saline and sweat from blue and some black inks is consistent with the presence of phthalocyanine‐based pigments, as suggested in the literature [18]. Cobalt and Ni extractions were variable but detectable in several inks. Although absolute concentrations were low, even minimal bioaccessible fractions are toxicologically relevant, as metal‐induced sensitization can occur at microgram‐level exposures. This finding aligns with clinical case reports of allergic reactions potentially triggered by Ni in tattoo pigments [37]. Metal solubility was strongly dependent on the extraction medium (Table 3 and Figure 2), reflecting the complex interactions between pigment matrices and biologically relevant fluids. As shown in Figure 2, deionized water generally yielded the lowest bioaccessible fractions for most toxicologically relevant elements such as As, Cd, Co, Cr and Hg. This behaviour is expected, as pure aqueous media lack the ionic strength and complexing capacity required to effectively mobilize elements from insoluble or strongly bound pigment matrices. In dissolution experiments on cosmetic pigments, Co and Ni showed negligible release in artificial sweat or simple aqueous media [21]. Although no specific studies on cosmetic pigments have compared dissolution in simple versus more chemically complex fluids, research in related systems (e.g., metal powders in simulated biological fluids) shows that the composition of the fluid can strongly influence metal release, with higher ion release observed in acidic or complexing simulated biological fluids compared with neutral media [38, 39]. In contrast, Cu and Zn exhibited comparatively higher bioaccessible fractions and greater variability among samples, suggesting that their pigment forms (e.g., phthalocyanine‐ or zinc oxide‐based) are more prone to partial dissolution even in low‐ionic‐strength media. A high prevalence and variability of Cu‐ and Zn‐containing pigments in commercial tattoo inks has also been reported in multi‐element surveys [7]. Physiological saline produced a marked increase in bioaccessibility for several elements—particularly Ba, Cr, Ni and Pb—indicating that higher ionic strength and the presence of chloride ions can enhance the dissolution of both matrix‐bound and nanoparticulate metals. This trend, evident across multiple ink colours in Figure 2, highlights the strong influence of extraction medium composition on metal mobilization. Artificial sweat generated intermediate solubility patterns, with Cu, Ni and Zn remaining among the most bioaccessible metals. These results indicate that dermal‐relevant fluids can facilitate the release of sensitizing or potentially toxic elements, as also observed in pigment dissolution studies employing artificial sweat [21]. Kruskal–Wallis tests (Table 3) revealed statistically significant differences among media for As, Ba, Cr, Ni, Se and Sb (*p* < 0.05), highlighting the necessity of evaluating multiple biofluids to obtain realistic exposure estimates. The dissolution patterns shown in Figure 2 illustrate how each medium selectively mobilizes different metals, underscoring the heterogeneous chemical behaviour of inorganic and organometallic pigment species. Although total concentrations of As, Cd, Pb and Sn in several inks exceeded the maximum permissible limits set by REACH Annex XVII (Figure 1 and Tables S5–S9), consistent with levels reported in recent surveys of European tattoo and permanent‐makeup inks [7], the fractions solubilized in biological fluids were generally much lower than the corresponding total contents. Nonetheless, low solubility should not be interpreted as absence of potential hazard. Recent studies have shown that metal oxide nanoparticles present in tattoo inks—particularly CuO and ZnO—can exhibit cytotoxic effects in vitro and are capable of penetrating human skin explants in ex vivo models [40]. However, such findings should be interpreted with caution, as in vitro and ex vivo results cannot be directly translated into in vivo risk, and differences in exposure conditions and concentrations limit direct comparison with the present study. Therefore, even modest bioaccessible fractions of sensitizing elements (e.g., Co, Cr, Ni) may warrant attention, although their contribution to adverse health effects under realistic exposure conditions remains to be fully elucidated. These considerations highlight the need for sensitive and standardized analytical workflows for the reliable assessment of metal release from tattoo inks.

**TABLE 3:** Levels of the elements measured in all ink samples after extraction in deionized water, physiological solution and artificial sweat.

	As	Ba	Cd	Co	Cr	Cu	Hg	Ni	Pb	Sb	Se	Sn	Zn
	Bioaccessible fraction in deionized water^a^												
**Mean**	0.015	0.9	0.007	0.0031	0.094	10	0.00112	0.114	0.021	0.052	0.022	0.0039	2.1
**SD**	0.014	1.9	0.022	0.0029	0.099	23	0.00041	0.066	0.064	0.054	0.043	0.0039	3.8
**Median**	< 0.01	0.04	0.001	0.0020	0.070	0.2	0.00110	0.100	0.003	0.024	< 0.01	0.0022	0.5
**Min**	< 0.01	< 0.03	< 0.00003	< 0.0002	< 0.03	< 0.01	0.00020	< 0.02	0.001	< 0.005	< 0.01	< 0.0002	< 0.2
**Max**	0.050	5.6	0.094	0.0110	0.430	97	0.00250	0.290	0.294	0.241	0.187	0.0143	15.6
	**Bioaccessible fraction in physiological saline solution** ^a^												
**Mean**	0.043	4.1	0.019	0.0042	0.36	1.3	0.00109	0.25	0.5	0.010	0.081	0.0052	6
**SD**	0.028	7.3	0.056	0.0032	0.31	2.5	0.00038	0.17	1.9	0.016	0.051	0.0057	14
**Median**	0.041	0.1	0.002	0.0033	< 0.3	0.1	0.00105	0.20	0.004	0.004	0.080	0.0037	0.5
**Min**	< 0.02	0.1	< 0.0004	< 0.0003	< 0.3	< 0.1	0.00025	0.05	< 0.003	0.0020	< 0.08	0.0006	< 0.3
**Max**	0.119	24.1	0.226	0.0118	1.20	8.8	0.00220	0.74	7.6	0.0583	0.280	0.0260	45
	**Bioaccessible fraction in artificial sweat** ^a^												
**Mean**	0.025	2.1	0.016	0.0045	0.21	1.8	0.00106	0.27	0.029	0.023	0.031	0.0040	7
**SD**	0.021	3.9	0.046	0.0032	0.20	3.3	0.00040	0.15	0.072	0.014	0.024	0.0075	13
**Median**	< 0.02	0.17	0.003	0.004	< 0.2	0.4	0.00100	0.23	< 0.01	0.019	< 0.04	0.0017	< 2
**Min**	< 0.02	< 0.03	< 0.002	< 0.002	< 0.2	< 0.07	0.00020	0.09	< 0.01	0.011	< 0.04	< 0.001	< 2
**Max**	0.083	14.1	0.187	0.0110	0.70	13.9	0.00210	0.62	0.310	0.071	0.110	0.0334	52
	***p*‐value** ^b^												
***p*_dw‐ps_**	***	**	ns	ns	***	ns	ns	***	ns	***	***	ns	ns
***p*_dw‐s_**	*	**	ns	*	**	ns	ns	***	ns	**	**	ns	*
***p*_ps‐s_**	**	*	ns	ns	**	ns	ns	ns	ns	***	***	*	ns

^a^ Levels are expressed in mg/kg.

^b^ *p*‐values from the Kruskal–Wallis test for independent samples and pairwise comparisons with Bonferroni correction for multiple testing are reported at the bottom of the table. *p*_dw–ps_ refers to the comparison between deionized water and physiological solution; *p*_dw–s_ to the comparison between deionized water and artificial sweat; and *p*_ps–s_ to the comparison between physiological solution and artificial sweat. ns: not significant (*p* > 0.05); *: significant at *p* < 0.05; **: significant at *p* < 0.01; and ***: significant at *p* < 0.001.

The combined assessment of total and soluble fractions provides a more complete picture of the potential exposure to metallic elements from tattoo inks. The results confirm large variability across commercial products, both in elemental profiles and in release behaviour, and demonstrate that extraction medium selection significantly affects the estimated soluble fraction. Standardization of bioaccessibility protocols will be essential to ensure consistent regulatory enforcement and comparability among laboratories.

## 4 Conclusions

The present study provides a comprehensive characterization of both total and bioaccessible metal contents in commercially available tattoo inks, using a validated workflow combining microwave‐assisted acid digestion and extractions in biologically relevant media. Considerable variability in total metal concentrations was observed across pigment types, particularly Ba in yellow inks, Cu in blue inks and Zn in several formulations, reflecting the diversity of pigment chemistries. Although several samples complied with current REACH limits, detectable levels of elements of toxicological concern, including As, Cd, Sn and Pb, were still observed, with total Pb concentrations exceeding regulatory thresholds in some ink colours. Despite the generally low bioaccessible fraction of Pb (≤ 2%), its presence at elevated total levels remains relevant from a regulatory and toxicological perspective. Total Cr concentrations were also frequently above regulatory thresholds; however, it should be noted that current regulations apply specifically to hexavalent chromium [Cr(VI)], and not to total chromium.

Bioaccessibility tests showed that only a fraction of the total metal content is soluble in deionized water, physiological saline, or artificial sweat, with significant differences among extraction media. Several elements, including As, Ba, Cr, Ni, Pb, Sb and Sn, exhibited solubility that was significantly dependent on the extraction medium, highlighting the importance of matrix‐specific bioaccessibility assessments. These findings underscore that bioaccessible fractions cannot be inferred from total contents alone, and that both the nature of the pigment and the composition of the extraction medium strongly influence metal mobilization.

The analytical workflow proved robust, providing accurate and precise quantification of both total and soluble metal fractions despite the complexity of tattoo ink matrices. These results emphasize the need for reliable, well‐validated analytical protocols and the adoption of standardized, chemically defined extraction media to improve reproducibility and comparability of bioaccessibility data. Integrating total content and bioaccessible fractions into risk assessments enhances the analytical basis for evaluating potential exposure and supports more consistent, mechanistically informed evaluations of tattoo ink safety. Although the primary focus of this study is human exposure assessment, the proposed analytical framework may also contribute to broader monitoring strategies for metal‐containing consumer products.

## Author Contributions

**Maria Luisa Astolfi**: conceptualization, methodology, validation, formal analysis, investigation, resources, data curation, writing – original draft, writing – review and editing, visualization, supervision. **Lorenzo Zara**: investigation, data curation. **Arianna Antonucci**: resources, data curation. **Carmela Protano**: conceptualization, resources, writing – review and editing, funding acquisition.

## Ethics Statement

The authors have nothing to report.

## Conflicts of Interest

The authors declare no conflicts of interest.

## Supporting information




**Supporting File**: ansa70103‐sup‐0001‐SuppMat.docx.

## Data Availability

Data will be made available on request.
