# Supplementary material for: Beyond Total Content: Assessing the Bioaccessibility of Metals in Tattoo Inks Using Validated Extraction Media
Source: Anal Sci Adv. 2026 Aug 7;7(2):e70103. doi: 10.1002/ansa.70103 (PMC13451626; doi:10.1002/ansa.70103)
Supplement: Supplementary file 1 [file ANSA-7-e70103-s001.docx]

Supplementary material

**Beyond total content: assessing the bioaccessibility of metals in tattoo inks using validated extraction media**

Maria Luisa Astolfi^a,*^, Lorenzo Zara^b^, Arianna Antonucci^c^, Carmela Protano^c^

^a^ Department of Chemistry, Sapienza University of Rome, 00185 Rome, Italy

^b^ MSD Animal Health, via Nettunense, km 20.300, 04011 Aprilia, Latina, Italy

^c^ Department of Public Health and Infectious Diseases, Sapienza University of Rome, 00185 Rome, Italy

Corresponding author.

E-mail address: marialuisa.astolfi@uniroma1.it (M.L. Astolfi).

**Table S1.** ICP-MS operating conditions applied to all tattoo ink samples, including digests prepared with HF, digests prepared without HF, and aqueous extracts. Instrumental parameters are reported for both operating modes, with and without the collision–reaction interface (CRI).

| ***Flow Parameters (L/min)*** | | | | |
| --- | --- | --- | --- | --- |
| Plasma Flow  Auxiliary Flow  Sheath Gas  Nebulizer Flow | 18.0  1.80  0.16  0.90 | | | |
| ***Other*** | | | | |
| Sampling Dept | 6.5 | | | |
| RF Power (kW) | 1.40 | | | |
| Pump Rate (rpm) | 3 | | | |
| Stabilization delay | 35 | | | |
| ***Samples with HF*** | | | ***Samples without HF*** | ***Samples with and without HF*** |
| ***Ion Optics (Volts)*** | | | | |
| First Extraction Lens | | -1 | -1 | -33 |
| Second Extraction Lens | | -106 | -55 | -165 |
| Third Extraction Lens | | -258 | -273 | -240 |
| Corner Lens | | -233 | -250 | -208 |
| Mirror Lens Left | | 37 | 37 | 30 |
| Mirror Lens Right | | 19 | 20 | 25 |
| Mirror Lens Bottom | | 52 | 47 | 28 |
| Entrance Lens | | 0 | 0 | 0 |
| Fringe Bias | | -3.2 | -3.7 | -2.4 |
| Entrance Plate | | -35 | -40 | -30 |
| Pole Bias | | 0.0 | 0.0 | 0.0 |
| ***CRI (mL/min)*** | | | | |
| Skimmer Gas Source | | OFF | OFF | H_2_ |
| Sampler Gas Source | | OFF | OFF | He |
| Skimmer Flow | | 0 | 0 | 70 |
| Sampler Flow | | 0 | 0 | 30 |

**Table S2.** CV-AFS operating conditions applied to all tattoo ink samples and aqueous extracts, except HF-prepared digests.

| **Parameter** | **Setting** |
| --- | --- |
| Photomultiplier tube high voltage | 275 V |
| Lamp current | 60 mA |
| Radiation source | Hg hollow-cathode lamp (253.7 nm) |
| Atomizer height | 10 mm |
| Quantification mode | Standard-curve quantification |
| Signal readout | Peak area |
| Read time | 15 s |
| Delay time | 0.5 s |
| Repetition time | 1 |
| Shield gas (Ar) flow rate | 1000 mL min⁻¹ |
| Carrier gas (Ar) flow rate | 300 mL min⁻¹ |

**Table S3.** Operating conditions for the microwave-assisted total acid digestion.

| **Step** | **Time**  **(min)** | **Temperature**  **(°C)** | **Maximum Power**  **(W)** |
| --- | --- | --- | --- |
| 1 | 5 | 0–50 | 1000 |
| 2 | 5 | 50 | 1000 |
| 3 | 5 | 50–100 | 1000 |
| 4 | 5 | 100 | 1000 |
| 5 | 5 | 100–150 | 1000 |
| 6 | 5 | 150 | 1000 |
| 7 | 5 | 150–180 | 1000 |
| 8 | 15 | 180 | 1000 |

**Table S4**. Detection and quantification limits, spike recoveries (R%, n = 6), and intra-day precision (as percent relative standard deviation, RSD%) for elements in fortified digested samples at two concentration levels.

|  |  |  |  | **Black ink** | | | | **White ink** | | | |
| --- | --- | --- | --- | --- | --- | --- | --- | --- | --- | --- | --- |
|  |  |  |  | **Level 1^a^** | | **Level 2^a^** | | **Level 1^a^** | | **Level 2^a^** | |
| **Element** | **REACH limits^b^** | **LOD^c^** | **LOQ^c^** | **R%** | **RSD%** | **R%** | **RSD%** | **R%** | **RSD%** | **R%** | **RSD%** |
| **As^d^** | 0.5 | 0.1 | 0.4 | 106 | 5 | 105 | 1 | 106 | 16 | 86 | 6 |
| **Ba** | 500 (soluble) | 1 | 2 | 95 | 1 | 91 | 15 | 104 | 5 | 99 | 6 |
| **Cd** | 0.5 | 0.07 | 0.02 | 107 | 1 | 110 | 2 | 96 | 4 | 97 | 1 |
| **Co** | 0.5 | 0.004 | 0.01 | 111 | 2 | 109 | 0.3 | 96 | 1 | 96 | 1 |
| **Cr^d,e^** | 0.5 | 0.4 | 1 | 112 | 11 | 108 | 15 | 115 | 13 | 93 | 10 |
| **Cu** | 250 (soluble) | 0.3 | 1 | 114 | 13 | 106 | 3 | 94 | 0.4 | 105 | 3 |
| **Hg^f^** | 0.5 | 0.0007 | 0.002 | 98 | 6 | 101 | 4 | 97 | 7 | 110 | 5 |
| **Ni** | 5 | 0.05 | 0.2 | 110 | 0.4 | 110 | 2 | 104 | 3 | 93 | 4 |
| **Pb** | 0.7 | 0.03 | 0.09 | 113 | 0.1 | 114 | 4 | 120 | 15 | 100 | 14 |
| **Sb** | 0.5 | 0.1 | 0.2 | 106 | 0.1 | 108 | 1 | 104 | 16 | 105 | 1 |
| **Se^d^** | 2 | 0.1 | 0.2 | 76 | 4 | 107 | 4 | 102 | 17 | 89 | 4 |
| **Sn** | 0.5 | 0.01 | 0.02 | 118 | 10 | 113 | 6 | 91 | 15 | 112 | 9 |
| **Zn** | 2000 (soluble) | 1 | 4 | 120 | 0.4 | 116 | 3 | 118 | 3 | 116 | 4 |

^a^Spikes 1 and 2 corresponded to 1/10 and 1/2 of the legal limit specified by REACH [1].

^b^ Limit concentrations are expressed in mg/kg.

^c^ Detection and quantification limits (LOD and LOQ; mg/kg).

^d^The collision–reaction interface (CRI) mode was applied for Cr, As, and Se to minimize polyatomic interferences.

^e^The limit is related to Cr(VI).

^f^Element determined by CV-AFS.

**Table S5.** Levels of the elements measured in black ink samples (n = 6) after microwave-assisted acid digestion and extraction in deionized water, physiological solution, and artificial sweat.

|  | **As** | **Ba** | **Cd** | **Co** | **Cr** | **Cu** | **Hg** | **Ni** | **Pb** | **Sb** | **Se** | **Sn** | **Zn** |
| --- | --- | --- | --- | --- | --- | --- | --- | --- | --- | --- | --- | --- | --- |
|  | *Total content by microwave-assisted acid digestion* | | | | | | | | | | | | |
| **Mean^a^** | <0.1 | <1 | <0.07 | <0.004 | 1.10 | 4.2 | 0.0038 | 0.48 | 6.9 | 0.31 | <0.1 | 0.40 | 6.5 |
| **SD^a^** | - | - | - | - | 0.65 | 3.9 | 0.0016 | 0.16 | 9.8 | 0.25 | - | 0.33 | 4.2 |
|  | *Bioaccessible fraction in deionized water* | | | | | | | | | | | | |
| **Mean^a^** | 0.012 | <0.03 | 0.030 | 0.0039 | 0.11 | 0.18 | 0.0013 | 0.13 | 0.09 | 0.11 | 0.082 | 0.0074 | 1.6 |
| **SD^a^** | 0.009 | - | 0.045 | 0.0036 | 0.06 | 0.14 | 0.0005 | 0.07 | 0.13 | 0.08 | 0.070 | 0.0057 | 2.0 |
| ***BF%^b^*** | *nd* | *nd* | *nd* | *nd* | *10* | *4* | *34* | *28* | *1* | *36* | *nd* | *2* | *25* |
|  | *Bioaccessible fraction in physiological saline solution* | | | | | | | | | | | | |
| **Mean^a^** | 0.039 | <0.1 | 0.07 | 0.0071 | <0.3 | <0.1 | 0.0012 | 0.30 | 0.10 | 0.004 | 0.100 | 0.009 | 2.2 |
| **SD^a^** | 0.029 | - | 0.11 | 0.0045 | - | - | 0.0007 | 0.19 | 0.06 | 0.002 | 0.094 | 0.012 | 2.8 |
| ***BF%^b^*** | *nd* | *nd* | *nd* | *nd* | *nd* | *nd* | *32* | *63* | *2* | *1* | *nd* | *2* | *33* |
|  | *Bioaccessible fraction in sweat* | | | | | | | | | | | | |
| **Mean^a^** | <0.02 | 0.091 | 0.063 | 0.0065 | <0.2 | 0.25 | 0.0011 | 0.28 | 0.11 | 0.017 | 0.061 | 0.012 | <2 |
| **SD^a^** | - | 0.091 | 0.094 | 0.0044 | - | 0.20 | 0.0008 | 0.19 | 0.04 | 0.004 | 0.041 | 0.015 | - |
| ***BF%^b^*** | *nd* | *nd* | *nd* | *nd* | *nd* | *6* | *29* | *59* | *2* | *5* | *nd* | *3* | *nd* |

^a^The levels, expressed as mean and standard deviation, are reported in mg/kg.

^b^The percentage of bioaccessible fraction (BF, %) in the aqueous extracts (deionized water, physiological saline, and artificial sweat) was calculated relative to the total content determined after complete acid digestion.

**Table S6**. Levels of the elements measured in white ink samples (n = 6) after microwave-assisted acid digestion and extraction in deionized water, physiological solution, and artificial sweat.

|  | **As** | **Ba** | **Cd** | **Co** | **Cr** | **Cu** | **Hg** | **Ni** | **Pb** | **Sb** | **Se** | **Sn** | **Zn** |
| --- | --- | --- | --- | --- | --- | --- | --- | --- | --- | --- | --- | --- | --- |
|  | *Total content by microwave-assisted acid digestion* | | | | | | | | | | | | |
| **Mean^a^** | 0.417 | 3.9 | 0.118 | <0.004 | 1.02 | 33.0 | 0.0023 | 0.81 | 4.75 | 0.295 | <0.1 | 1.77 | 266 |
| **SD^a^** | 0.058 | 3.3 | 0.004 | - | 0.03 | 0.5 | 0.0021 | 0.01 | 0.05 | 0.002 | - | 0.03 | 167 |
|  | *Bioaccessible fraction in deionized water* | | | | | | | | | | | | |
| **Mean^a^** | 0.017 | <0.03 | 0.0018 | 0.0011 | 0.064 | 0.20 | 0.0017 | 0.094 | 0.0036 | 0.041 | <0.007 | 0.0031 | 5.2 |
| **SD^a^** | 0.013 | - | 0.0016 | 0.0005 | 0.040 | 0.20 | 0.0005 | 0.038 | 0.0024 | 0.031 | - | 0.0040 | 6.8 |
| ***BF%^b^*** | *4* | *nd* | *2* | *nd* | *6* | *1* | *72* | *12* | *0.08* | *14* | *nd* | *0.2* | *2* |
|  | *Bioaccessible fraction in physiological saline solution* | | | | | | | | | | | | |
| **Mean^a^** | <0.02 | <0.1 | 0.0054 | 0.0012 | <0.3 | <0.1 | 0.0016 | 0.10 | 0.0038 | 0.021 | <0.08 | 0.0036 | 27 |
| **SD^a^** | - | - | 0.0048 | 0.0012 | - | - | 0.0009 | 0.05 | 0.0042 | 0.027 | - | 0.0009 | 21 |
| ***BF%^b^*** | *nd* | *nd* | *5* | *nd* | *nd* | *nd* | *70* | *13* | *0.08* | *7* | *nd* | *0.2* | *10* |
|  | *Bioaccessible fraction in sweat* | | | | | | | | | | | | |
| **Mean^a^** | 0.037 | <0.03 | 0.0043 | <0.002 | <0.2 | 0.49 | 0.0017 | 0.16 | <0.01 | 0.034 | <0.04 | 0.0011 | 24 |
| **SD^a^** | 0.026 | - | 0.0043 | - | - | 0.56 | 0.0011 | 0.05 | - | 0.028 | - | 0.0008 | 21 |
| ***BF%^b^*** | *9* | *nd* | *4* | *nd* | *nd* | *1* | *72* | *19* | *nd* | *11* | *nd* | *0.06* | *9* |

^a^The levels, expressed as mean and standard deviation, are reported in mg/kg.

^b^The percentage of bioaccessible fraction (BF, %) in the aqueous extracts (deionized water, physiological saline, and artificial sweat) was calculated relative to the total content determined after complete acid digestion.

**Table S7.** Levels of the elements measured in yellow ink samples (n = 6) after microwave-assisted acid digestion and extraction in deionized water, physiological solution, and artificial sweat.

|  | **As** | **Ba** | **Cd** | **Co** | **Cr** | **Cu** | **Hg** | **Ni** | **Pb** | **Sb** | **Se** | **Sn** | **Zn** |
| --- | --- | --- | --- | --- | --- | --- | --- | --- | --- | --- | --- | --- | --- |
|  | *Total content by microwave-assisted acid digestion* | | | | | | | | | | | | |
| **Mean^a^** | 0.50 | 3930 | 1.51 | 0.152 | 6 | 6.2 | 0.0049 | 1.76 | 0.216 | 0.23 | <0.1 | <0.01 | 8360 |
| **SD^a^** | 0.90 | 297 | 0.01 | 0.001 | 12 | 1.4 | 0.0045 | 0.12 | 0.018 | 0.39 | - | - | 180 |
|  | *Bioaccessible fraction in deionized water* | | | | | | | | | | | | |
| **Mean^a^** | <0.01 | 3.6 | 0.0005 | 0.0027 | 0.062 | 0.20 | 0.0012 | 0.12 | 0.0024 | 0.021 | <0.007 | 0.0020 | 2.6 |
| **SD^a^** | - | 2.8 | 0.0002 | 0.0015 | 0.026 | 0.21 | 0.0007 | 0.06 | 0.0017 | 0.002 | - | 0.0008 | 3.9 |
| ***BF%^b^*** | *nd* | *0.09* | *0.03* | *2* | *1* | *3* | *24* | *7* | *1* | *9* | *nd* | *nd* | *0.03* |
|  | *Bioaccessible fraction in physiological saline solution* | | | | | | | | | | | | |
| **Mean^a^** | 0.053 | 13 | 0.0010 | 0.0041 | 0.36 | <0.1 | 0.0012 | 0.26 | 0.0046 | 0.0030 | <0.08 | 0.0034 | 0.94 |
| **SD^a^** | 0.033 | 11 | 0.0006 | 0.0021 | 0.24 | - | 0.0008 | 0.10 | 0.0037 | 0.0011 | - | 0.0006 | 0.94 |
| ***BF%^b^*** | *11* | *0.3* | *0.06* | *3* | *6* | *nd* | *24* | *15* | *2* | *1* | *nd* | *nd* | *0.01* |
|  | *Bioaccessible fraction in sweat* | | | | | | | | | | | | |
| **Mean^a^** | 0.026 | 6.9 | <0.002 | 0.0050 | 0.23 | 0.38 | 0.0011 | 0.27 | <0.01 | 0.018 | <0.04 | 0.0016 | 4.9 |
| **SD^a^** | 0.014 | 5.9 | - | 0.0030 | 0.29 | 0.19 | 0.0010 | 0.11 | - | 0.004 | - | 0.0007 | 6.1 |
| ***BF%^b^*** | *5* | *0.2* | *nd* | *3* | *4* | *6* | *22* | *15* | *nd* | *8* | *nd* | *nd* | *0.06* |

^a^The levels, expressed as mean and standard deviation, are reported in mg/kg.

^b^The percentage of bioaccessible fraction (BF, %) in the aqueous extracts (deionized water, physiological saline, and artificial sweat) was calculated relative to the total content determined after complete acid digestion.

**Table S8.** Levels of the elements measured in red ink samples (n = 6) after microwave-assisted acid digestion and extraction in deionized water, physiological solution, and artificial sweat.

|  | **As** | **Ba** | **Cd** | **Co** | **Cr** | **Cu** | **Hg** | **Ni** | **Pb** | **Sb** | **Se** | **Sn** | **Zn** |
| --- | --- | --- | --- | --- | --- | --- | --- | --- | --- | --- | --- | --- | --- |
|  | *Total content by microwave-assisted acid digestion* | | | | | | | | | | | | |
| **Mean^a^** | <0.1 | 52.3 | 0.025 | 0.0910 | 2.13 | 4.38 | 0.0051 | 1.79 | 0.887 | 0.43 | <0.1 | 0.351 | 4.2 |
| **SD^a^** | - | 2.5 | 0.001 | 0.0081 | 0.60 | 0.78 | 0.0019 | 0.08 | 0.012 | 0.65 | - | 0.018 | 2.1 |
|  | *Bioaccessible fraction in deionized water* | | | | | | | | | | | | |
| **Mean^a^** | 0.017 | 0.8 | 0.00051 | 0.0041 | 0.077 | 0.24 | 0.0006 | 0.15 | 0.0047 | 0.064 | <0.007 | 0.0037 | 0.4 |
| **SD^a^** | 0.013 | 1.0 | 0.00030 | 0.0043 | 0.028 | 0.18 | 0.0002 | 0.09 | 0.0056 | 0.051 | - | 0.0033 | 0.4 |
| ***BF%^b^*** | *nd* | *1* | *2* | *5* | *4* | *5* | *11* | *8* | *1* | *15* | *nd* | *1* | *9* |
|  | *Bioaccessible fraction in physiological saline solution* | | | | | | | | | | | | |
| **Mean^a^** | 0.037 | 4.4 | 0.0006 | 0.0034 | <0.3 | <0.1 | 0.0006 | 0.31 | <0.003 | 0.0041 | <0.08 | 0.0032 | <0.3 |
| **SD^a^** | 0.017 | 6.5 | 0.0002 | 0.0019 | - | - | 0.0005 | 0.26 | - | 0.0008 | - | 0.0020 | - |
| ***BF%^b^*** | *nd* | *8* | *2* | *4* | *nd* | *nd* | *11* | *17* | *nd* | *0.9* | *nd* | *0.9* | *nd* |
|  | *Bioaccessible fraction in sweat* | | | | | | | | | | | | |
| **Mean^a^** | <0.02 | 2.5 | 0.0050 | 0.0033 | <0.2 | 0.29 | 0.0006 | 0.32 | <0.01 | 0.019 | <0.04 | 0.0023 | <2 |
| **SD^a^** | - | 3.6 | 0.0074 | 0.0020 | - | 0.14 | 0.0006 | 0.18 | - | 0.004 | - | 0.0016 | - |
| ***BF%^b^*** | *nd* | *5* | *20* | *4* | *nd* | *7* | *11* | *18* | *nd* | *4* | *nd* | *0.7* | *nd* |

^a^The levels, expressed as mean and standard deviation, are reported in mg/kg.

^b^The percentage of bioaccessible fraction (BF, %) in the aqueous extracts (deionized water, physiological saline, and artificial sweat) was calculated relative to the total content determined after complete acid digestion.

**Table S9.** Levels of the elements measured in blue ink samples (n = 6) after microwave-assisted acid digestion and extraction in deionized water, physiological solution, and artificial sweat.

|  | **As** | **Ba** | **Cd** | **Co** | **Cr** | **Cu** | **Hg** | **Ni** | **Pb** | **Sb** | **Se** | **Sn** | **Zn** |
| --- | --- | --- | --- | --- | --- | --- | --- | --- | --- | --- | --- | --- | --- |
|  | *Total content by microwave-assisted acid digestion* | | | | | | | | | | | | |
| **Mean^a^** | 0.90 | 11.2 | 0.104 | 0.170 | 1.89 | 17700 | 0.0019 | 4.0 | 4.32 | 0.264 | <0.1 | 1.59 | 107 |
| **SD^a^** | 0.45 | 7.2 | 0.020 | 0.055 | 0.78 | 5810 | 0.0021 | 0.4 | 0.08 | 0.005 | - | 0.01 | 1 |
|  | *Bioaccessible fraction in deionized water* | | | | | | | | | | | | |
| **Mean^a^** | 0.024 | 0.11 | 0.0020 | 0.0034 | 0.16 | 51 | 0.0009 | 0.068 | 0.0053 | 0.024 | 0.008 | 0.0032 | 0.84 |
| **SD^a^** | 0.022 | 0.07 | 0.0017 | 0.0027 | 0.21 | 40 | 0.0003 | 0.046 | 0.0048 | 0.019 | 0.012 | 0.0025 | 0.76 |
| ***BF%^b^*** | *3* | *1* | *2* | *2* | *8* | *0.3* | *47* | *2* | *0.1* | *9* | *nd* | *0.2* | *0.8* |
|  | *Bioaccessible fraction in physiological saline solution* | | | | | | | | | | | | |
| **Mean^a^** | 0.069 | 2.4 | 0.013 | 0.0051 | 0.71 | 6.0 | 0.0009 | 0.27 | <0.003 | 0.018 | 0.093 | 0.0072 | 0.86 |
| **SD^a^** | 0.022 | 3.2 | 0.022 | 0.0024 | 0.46 | 1.6 | 0.0006 | 0.12 | - | 0.019 | 0.031 | 0.0027 | 0.70 |
| ***BF%^b^*** | *8* | *21* | *12* | *3* | *37* | *0.03* | *47* | *7* | *nd* | *7* | *nd* | *0.5* | *0.8* |
|  | *Bioaccessible fraction in sweat* | | | | | | | | | | | | |
| **Mean^a^** | 0.030 | 0.83 | 0.0029 | 0.0061 | 0.23 | 7.7 | 0.0009 | 0.35 | <0.01 | 0.027 | <0.04 | 0.0032 | <2 |
| **SD^a^** | 0.029 | 0.69 | 0.0004 | 0.0022 | 0.18 | 3.6 | 0.0008 | 0.17 | - | 0.008 | - | 0.0019 | - |
| ***BF%^b^*** | *3* | *7* | *3* | *4* | *12* | *0.04* | *47* | *9* | *nd* | *10* | *nd* | *0.2* | *nd* |

^a^The levels, expressed as mean and standard deviation, are reported in mg/kg.

^b^The percentage of bioaccessible fraction (BF, %) in the aqueous extracts (deionized water, physiological saline, and artificial sweat) was calculated relative to the total content determined after complete acid digestion.

**References**

[1] Commission Regulation (EU) 2020/2081 of 14 December 2020 amending Annex XVII to Regulation (EC) No 1907/2006 of the European Parliament and of the Council concerning the registration, evaluation, authorisation and restriction of chemicals (REACH) as regards substances in tattoo inks or permanent make-up, Official Journal of the European Union 423 (2020): 6–17. https://eur-lex.europa.eu/eli/reg/2020/2081/oj (accessed 19 November 2025).
